# What Is Grazing Time? Insights from the Acoustic Signature of Goat Jaw Activity in Wooded Landscapes

**DOI:** 10.3390/s25010008

**Published:** 2024-12-24

**Authors:** Eugene David Ungar, Reuven Horn

**Affiliations:** 1 Department of Natural Resources, Institute of Plant Sciences, Agricultural Research Organization (ARO), Volcani Center, 68 HaMaccabim Road, P.O. Box 15159, Rishon LeZion 7505101, Israel; 2 Rangeland Service, Ministry of Agriculture and Food Security, P.O. Box 30, Rishon LeZion 5025001, Israel; reuvenho@moag.gov.il

**Keywords:** foraging, grazing behavior, herbaceous vegetation, intake, Mediterranean, rumination, shrubs, signal processing

## Abstract

Acoustic monitoring facilitates the detailed study of herbivore grazing by generating a timeline of sound bursts associated with jaw movements (JMs) that perform bite or chew actions. The unclassified stream of JM events was used here in an observational study to explore the notion of “grazing time”. Working with shepherded goat herds in a wooded landscape, a horn-based acoustic sensor with a vibration-type microphone was deployed on a volunteer animal along each of 12 foraging routes. The software-generated timeline of unclassified JMs contained a total of 334,582 events. After excluding rumination bouts, minutely JM rates showed a broad, non-normal distribution, with an overall mean of 61 JM min^−1^. The frequency distribution of inter-JM interval values scaled logarithmically, with a peak in the region of 0.43 s representing a baseline interval that generates the unconstrained, more-or-less regular, rhythm of jaw movement (≈140 JM min^−1^). This rhythm was punctuated by interruptions, for which duration scaled logarithmically, and which were primarily related to the search phase of the intake process. The empirical time accumulation curve shows the contribution of the inter-JM interval to the total foraging time and provides a penetrating profile of how the animal interacted with the foraging environment. The sum total of time along a foraging route spent at a near-potential JM rate was only ≈1 h, whereas sub-potential rates containing intervals as long as ≈30 s accounted for the bulk of the foraging route. The dimensionless behavioral grazing intensity was defined as the product of the number of ingestive JMs performed and the baseline interval, divided by the duration of the foraging route (excluding rumination). Values were mostly <0.5 for the foraging routes examined. This has implications for how animal presence should be translated to grazing pressure and for how long animals need to forage to meet their nutritional requirements.

## 1. Introduction

Despite being a globally important biological process, herbivore grazing is not studied routinely at a mechanistic level, perhaps because of a lack of suitable tools. The quantitative study of the grazing impacts of herbivore herds on natural landscapes requires *some* expressions of how much grazing/browsing took place and, at its simplest, that boils down to the number of mouths present, the duration of presence, and the total area available. The unit of measure might be animal-days of presence accumulated over a defined period (e.g., annually), per unit area available. But the area available can be tricky to define when dealing with open (unfenced) rangeland or even when dealing with fenced rangeland if the paddocks are extremely large. Furthermore, the more complex the landscape in terms of topography and vegetation, the more spatially heterogeneous are the grazing impacts expected to be [1,2].

To account for that heterogeneity, as well as to contend with the above “open-rangeland problem”, the next level of resolution expresses how much grazing took place in a spatially explicit way, essentially substituting the “area exploited” for the “area available”. In principle, this can be achieved by combining animal-borne GPS and GIS (e.g., [3]). In practice, this is challenging when studying herds that splinter into subgroups and even individuals, which may forage tens or hundreds of meters apart [4], and the age of being able to deploy a GPS collar on every animal in a herd has yet to dawn. However, an important subset of open rangelands is exploited by shepherded small-ruminant herds that, for a portion of the day, traverse the landscape along a meandering path as a fairly coherent unit. At any moment, the herd has an approximately rounded footprint area with a definable boundary, and there are ways of estimating it [5]. When a herd is tracked using only one GPS collar, the simplifying assumption is made that the GPS location of the volunteer animal is at the center of the footprint circle. It is possible, in principle, to ascribe to the patch of the landscape under the herd footprint (centered at the GPS location) the product of the number of mouths and (preferably short) GPS fix interval. By accumulating these values for all GPS locations over an entire season or year, one obtains a map of what can loosely be termed grazing pressure, with units of animal-days per unit area (also termed “stocking rate”) [6].

The next level of resolution in expressing how much grazing is taking place is to account for eating not being distributed evenly over time. This is especially relevant for free-ranging animals, which spend many hours in the course of a day resting in place. But it may also be relevant for small-ruminant herds corralled at night and shepherded by day, often for a limited number of hours. A reasonable working assumption would be that the animal grazes “all the time” because access time to the forage resource is being limited. But that assumption merits closer examination. First, it might be argued that the limited abundance and low nutritional quality of the rangeland vegetation constrains the expression of eating. Furthermore, the fact that the herd is guided along a route, rather than being left to forage and disperse naturally, may diminish the ability to be consistently engaged in eating and could conceivably give rise to an internal structure of discrete, separated bouts of eating and travelling.

Addressing these issues raises the more fundamental question as to what precisely constitutes being engaged in grazing. Traditionally, when conducting behavioral observations of individual animals, an interruption in eating-related activity that exceeds a certain threshold (e.g., 1 min) is considered “not-grazing” (e.g., [7,8]). The first problem is that the threshold is somewhat arbitrary. Secondly, a binary definition of grazing makes no distinction between, at one extreme, sporadic jaw activity with frequent below-threshold interruptions, and, at the other extreme, bouts of uninterrupted jaw activity typical of abundant, high-quality herbaceous swards; they are regarded equally as “grazing” [9].

To address these problems, we studied the detailed timeline of jaw-movement events (biting or chewing) of individual animals along a foraging route. Direct observations struggle to provide this timeline, not least because the orientation of the animal’s head and/or intervening vegetation will often obscure the line of sight. Furthermore, visually identifying and counting chewing jaw movements is more error prone than identifying the removal of a bite. Acoustic monitoring solves these problems and potentially provides a much more accurate and complete timeline of sound-generating jaw movements (biting and chewing actions) than can be achieved by direct observation [10]. For large datasets, automation of the sequencing task is essential, and various approaches have been reported (e.g., [11,12]). Contrary to studies in which the focus is on the bite rate, which require the classification of sound bursts into bites, chews and chew-bites (where relevant) [13], here it is imperative to include bite *and* chew actions in accounting for the grazing time, and hence, no classification is needed. This makes the signal-processing task easier and more accurate. A previously published algorithm was used to analyze the recordings and generate the timeline of unclassified jaw movements.

The general objective of this observational study was to characterize the behavior of shepherded goat herds foraging in a hilly, wooded environment with diverse vegetation, using the timeline of jaw activity furnished by acoustic monitoring. More specifically, the study sought to develop a non-binary method of defining grazing time that retains and portrays the fine structure of the foraging process.

## 2. Materials and Methods

### 2.1. Study Area

The study was conducted in the Judean Hills area, south-west of Jerusalem, with an average elevation of 650 m a.s.l. The soils of the region are mainly of marine sedimentary rock: chalk, dolomite, limestone, and marl. Predominantly, a low-limestone terra rossa soil was formed, but dark and light rendzina soils can also be found [14]. The area has a semi-humid Mediterranean climate with an average precipitation ranging from 400 to 600 mm yr^−1^ that falls during the 5–6 winter months (November–March/April). The 2013/2014 season in which the study was conducted was at the low end of the rainfall range (434 mm). The mean annual temperature is approximately 17 °C. The difference between the mean temperatures of the warmest and coldest months of the year is 15 °C. The mean relative humidity in the area is about 60% (Israel Meteorological Service, Beit Dagan, Israel; https://ims.gov.il/en, accessed on 19 December 2024).

### 2.2. Vegetation

The natural vegetation of the Judean Hills comprises mainly woodland, tall shrubs, dwarf shrubs, old stands of *Pinus halepensis* Mill., and a sparse undergrowth of herbaceous, including geophytic, vegetation. The most common natural woodlands in the Judean hills are communities dominated by *Quercus coccifera* L. and *Pistacia palaestina* L., accompanied by *Phillyrea latifolia* L., *Crataegus aronia* L., *Pistacia lentiscus* L., *Styrax officinalis* L., and *Ceratonia siliqua* L. The trees are accompanied by various species of woodland climbers. The primary tall-shrub communities of the Judean Hills are dominated by *Calicotome villosa* (Poir.) Link and *Rhamnus lycioides* L. The primary undergrowth communities are dominated by *Sarcopoterium spinosum* (L.) Spach and *Avena sterilis* L., accompanied by numerous annual herbaceous species [15].

Besides the natural vegetation in the Judean Hills, there are substantial areas of planted forests, established over the last century, consisting mainly of three coniferous pine species—*Pinus halepensis* Mill., *Pinus brutia* Ten., *Pinus canariensis* C.Sm. ex DC, and *Cupressus sempervirens* L.

### 2.3. Herds and Their Management

The study region as a whole was populated by seven mixed-age goat herds, which derived their primary source of nutrition from the vegetation and were shepherded across the landscape on a daily basis. Over a number of years, the daily foraging routes of these herds were monitored using GPS collars, and the data were converted into a map of cumulative grazing-days per unit area [6]. Six of these herds were used in the present study, each identified by a three-letter code. Two of the herds (YAR and HIM) were dairy herds; breeds were Mamber and Damascus for YAR and Mamber and Alpine for HIM. Herds YAR and HIM were based at permanent homesteads in the forest and raised primarily for cheese production. Kidding was in the months of December–March. The other four herds (KDE, REF, KDW, and MAT) were meat-breed herds, predominantly Damascus goats, with kidding in the months of October–November. These herds were introduced to the region by the forest management authority to suppress (by grazing and trampling) the undergrowth vegetation along strategic routes and fire buffer zones, thereby reducing the intensity and rate of spread of any encroaching fire. These herds were corralled at night in temporary encampments, which were relocated periodically through the annual cycle to impact different regions of the study area. The foraging routes of the “permanent” (dairy) herds tended to be of a shorter duration and uninterrupted, whereas those of the “mobile” herds could span most or all daylight hours, and included a mid-route resting and watering period in the hot summer months. High-quality supplementary feed was provided to the dairy herds during milking. The meat-breed herds received supplementary feed when vegetation abundance was low, after returning to the night corral [16]. The acoustic signal analyzed never included the consumption of supplementary feed.

### 2.4. Acoustic Signal Acquisition

Acoustic monitoring was performed using a small MP3 device (Sansa Clip+, Sandisk, Milpitas, CA, USA), modified to connect externally to a vibration-type microphone (Cherub WCP-55, Cherub-technology, Shenzhen, China). When properly attached to one horn of the animal, the signal recorded is that of sounds/vibrations that are transmitted throughout the hard tissues of the skull and horns when the animal performs jaw movements of a biting or chewing nature. Being a vibration-type microphone, it detects minimal background noise. The recording device is of low weight (24 g) and dimensions (178 × 127 × 31 mm) and has a slot for a MicroSD memory card (32 GB in this study). The same physical device was used throughout. To add critical functionality, we installed RockBox (https://www.rockbox.org/, accessed on 19 December 2024) on the device. This enabled files to be written to the memory card and allowed for the seamless, automatic closing and opening of files of a defined size (set to 500 MB) to enable continuous recording over many hours. Files were recorded in WAV format at a sampling frequency of 24 kHz, somewhat lower than the frequency used in an earlier study with the same sensor configuration [17], but without an aurally detectable loss of sound quality.

### 2.5. Acoustic Signal Processing

Given the large quantity of audio signals gathered, it was essential to automate the signal processing. As preparatory steps, the sound files underwent pre-processing using Audacity 2.0.5 (Audacity Team, 2013; https://www.audacityteam.org accessed on 19 December 2024), including noise reduction (applying default settings in conjunction with a 10 s inter-jaw-movement interval as the noise profile), signal filtering (60 Hz high-pass), and adjustment of the sampling rate in the file header based on the pre- and post-recording taps. The signal was then down-sampled to 8 kHz, which retains ample information to detect jaw movements while reducing the computational load. The identification of sound-generating jaw movements (of any type) was performed using an algorithm developed for this purpose by Navon et al. [17]. The software uses a set of criteria to differentiate between extraneous noises and true jaw movements and generates the timestamp of every jaw movement identified throughout the foraging route. To monitor the quality of the output, the timestamps were overlaid onto the acoustic signal waveform using Sonic Visualiser v2.1 [18], and at least 20 five-minute validation segments per foraging route were scanned for false positive and false negative events.

### 2.6. Deployment

**Figure 1:**
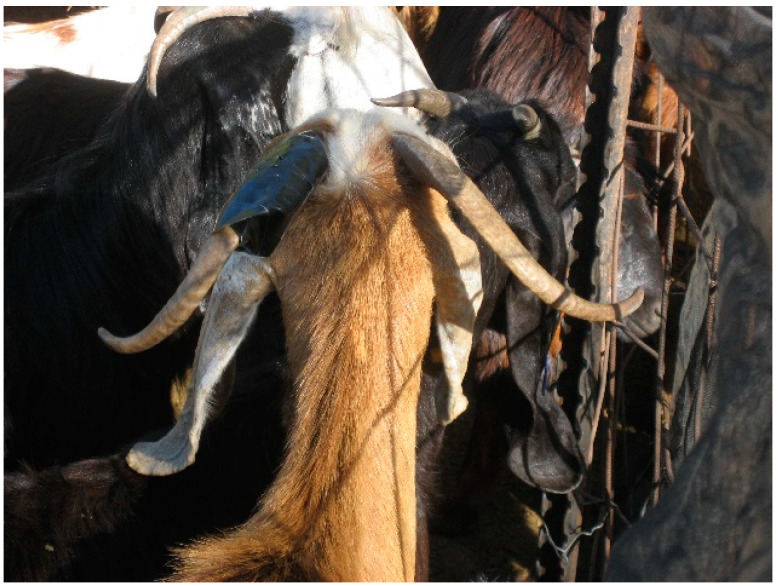
Photograph of goat with acoustic sensor attached to left horn. Taken on 6 April 2014, in the holding pen of herd KDE prior to commencement of the foraging route.

Acoustic monitoring was conducted on 12 dates between September 2013 and April 2014, half in the dry season and half in the green season. Herds KDW, MAT, REF, and YAR were monitored once in each season, herd KDE was monitored once in the dry season and twice in the wet season, and herd HIM was monitored once in the dry season only. Animals were predominantly in mid-to-late gestation in the dry season and in late gestation to early lactation in the green season. At each deployment, a volunteer animal was selected randomly from the herd. The recording device was placed inside a protective casing, and the entire assembly was attached to the smooth, inside face of one horn, with the microphone facing inwards, using duct tape (Figure 1). The sensor was attached to the horn approximately 30 min prior to the start of the foraging route for acclimation. To adjust for clock drift, a series of taps was recording at the beginning and end of each day’s recording at a precisely known time.

### 2.7. Data Processing

The timeline of unclassified jaw movement events was examined in two ways: time-based and event-based. In the time-based approach, JM events were summarized as counts within minutely slices and zero-padded for “quiet” minutes. In the event-based approach, the timeline was converted to a set of time intervals between consecutive JM events. These values were graphically displayed in sets of 500 values arrayed in chronological order and visually scanned for any distinctive patterns, such as that of rumination. In round numbers, 500 was the largest number of events that could be displayed at once while retaining sufficient resolution to distinguish individual events. We examined the length of runs of sequential interval values that remain below defined thresholds. The frequency distribution of the inter-jaw-movement interval was examined, as was that of the minutely rate of jaw movement (RJM). In addition, the interval values were examined as CDF plots and as the cumulative ascending ranked interval. These different perspectives on the raw data of unclassified jaw movement events were examined jointly to develop an approach to the notion of grazing time.

## 3. Results

### 3.1. Performance of the Acoustic Sensor

**Figure 2:**
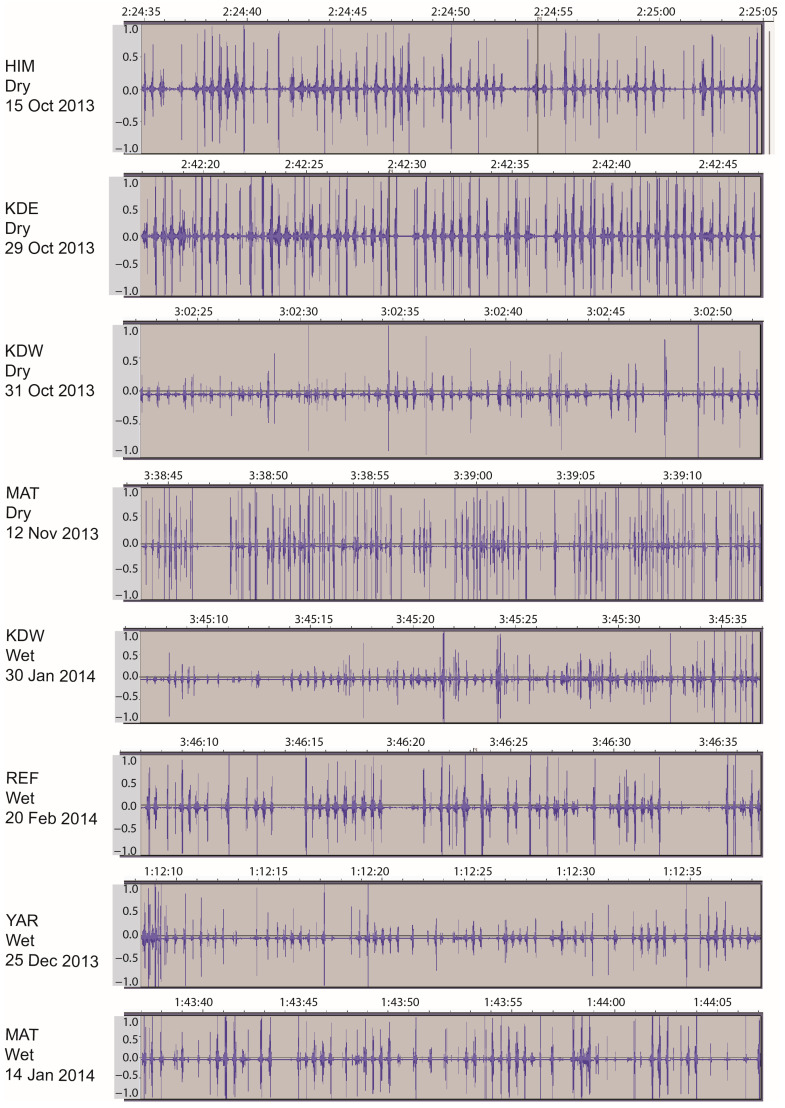
Examples of the waveform obtained via the acoustic monitoring of grazing goats in a Mediterranean shrubby and woody rangeland. The waveforms are a graphical representation of the pattern of sound pressure variation (amplitude) in the time domain. Each panel shows a 30 s segment of relatively active jaw activity, identified by herd (3-letter code defined in Table 1), season (wet or dry), and date. Times are hh:mm:ss.

The acoustic monitoring equipment did not disturb the animal in any observable way and its deployment between the two vertically oriented horns of a goat took advantage of a region that is relatively well-protected physically. The inner face of the horn provided a hard, smooth surface for close, firm contact by the microphone head, and in all 12 deployments, the vibration-type microphone yielded a high-quality signal with low background noise. When viewed at the appropriate scale, the sound bursts are clearly visible in the waveform of the acoustic signal, examples of which are shown in Figure 2. There was no aurally detectable effect of season on the nature of the sounds generated or on the visual qualities of the waveform. In general, chew actions generated a stronger signal than did bite actions and were easier to identify aurally. The opposite would be expected for the consumption of dense, high-quality herbaceous vegetation, which also generates more easily distinguishable sounds between bites and chews than obtained in this study.

### 3.2. Quality of the Signal Processing

The high quality of the sound signal enabled the timeline of sound bursts generated by jaw movements to be specified by the algorithm of Navon et al. [17] with high accuracy, as verified by the validation segments. The incidence of false positive (≈3%) and false negative (≈4%) events was low and well within the limits achieved in the original development of the algorithm [17]. The validation segments confirmed that noises, such as those caused by contact of the horn with vegetation, were identified as such by the algorithm and were not registered as jaw movements (any true jaw movements that may have co-occurred would not be registered).

### 3.3. Overview of the Acoustic Recordings

The total recording time analyzed was 91 h and a total of 334,582 jaw movement events were identified (Table 1), implying an overall average RJM of 61 min^−1^. In the course of visually scanning the waveforms and the event-based plots, a characteristic pattern, which could be interpreted only as rumination, appeared at least once along 10 of the 12 foraging routes. The two routes with no rumination were of the two dairy herds monitored in the dry season (YAR and HIM); the only dairy herd monitored in the wet season (YAR) had a small amount of “uncertain” rumination (Table 1).

### 3.4. The Pattern of Rumination Jaw Activity

**Figure 3:**
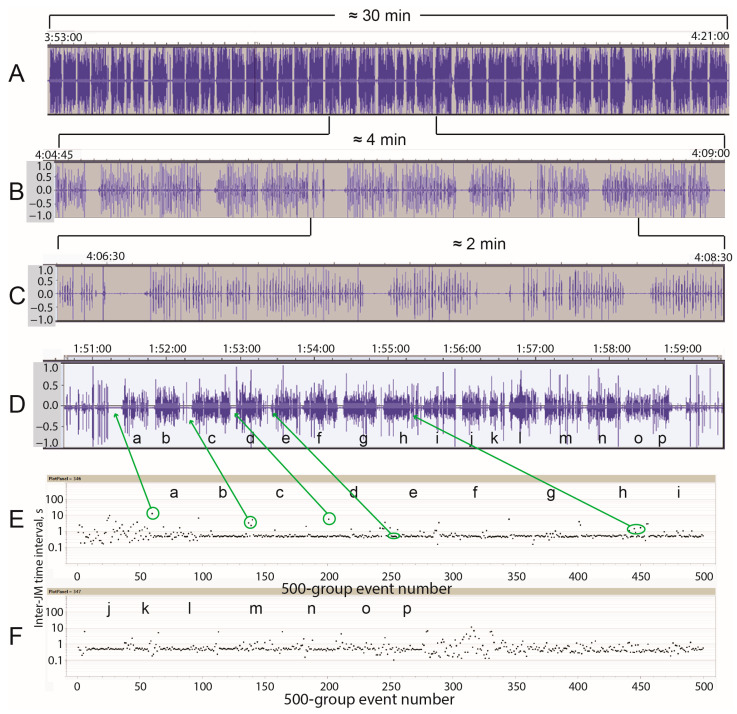
Rumination patterns of jaw movement from time-based and event-based perspectives. Panels (**A**–**C**) are waveforms of the sound signal taken from one foraging route (KDE, Dry, 29 October 2013) at different scales of enlargement spanning 30, 4, and 2 min, respectively. Panel (**D**) shows the waveform of a 7 min bout of rumination from a different foraging route (KDE, Wet, 6 April 2014); panels (**E**,**F**) show the corresponding event-based plot; corresponding boli have matching lower-case letters.

Rumination displayed a distinctive pattern of jaw movement events. When the field of view contains the waveform of an entire rumination bout, as shown in Figure 3A, the pattern comprises sustained, dense runs of sound bursts with similar amplitudes, studded at regular intervals by a small break. The time scale of the break (5–10 s) is consistent with the time required to swallow and regurgitate [19]. Compared to grazing, this pattern of jaw movement is machine-like. But a closer examination of Figure 3A reveals deviations and irregularities in the pattern of bolus duration and inter-bolus interval that might be expected when behavior, and not just biomechanics, is involved. At greater enlargement, the internal structure of individual boluses can be resolved to the chew level (Figure 3B,C). At this scale, the pattern is created by the following: (a) a rhythm of jaw movement within each bolus that is highly regular and similar across boluses; (b) the number of jaw movements per bolus being fairly consistent; and (c) a fairly consistent inter-bolus interval. But because of the variability in these and large variability in the duration of a rumination bout, from just a few boluses to many multiples of that, boundary cases arise that are ultimately judgement calls (the “uncertain” designation for rumination was used in such cases). An example of the waveform of a rumination bout with its corresponding event-based plot is shown in Figure 3D–F. Importantly, small irregularities and mini-pauses in the rhythm that are evident in the waveform (and the soundtrack) were tracked well by the algorithm and are reflected in the event-based plot.

**Figure 4:**
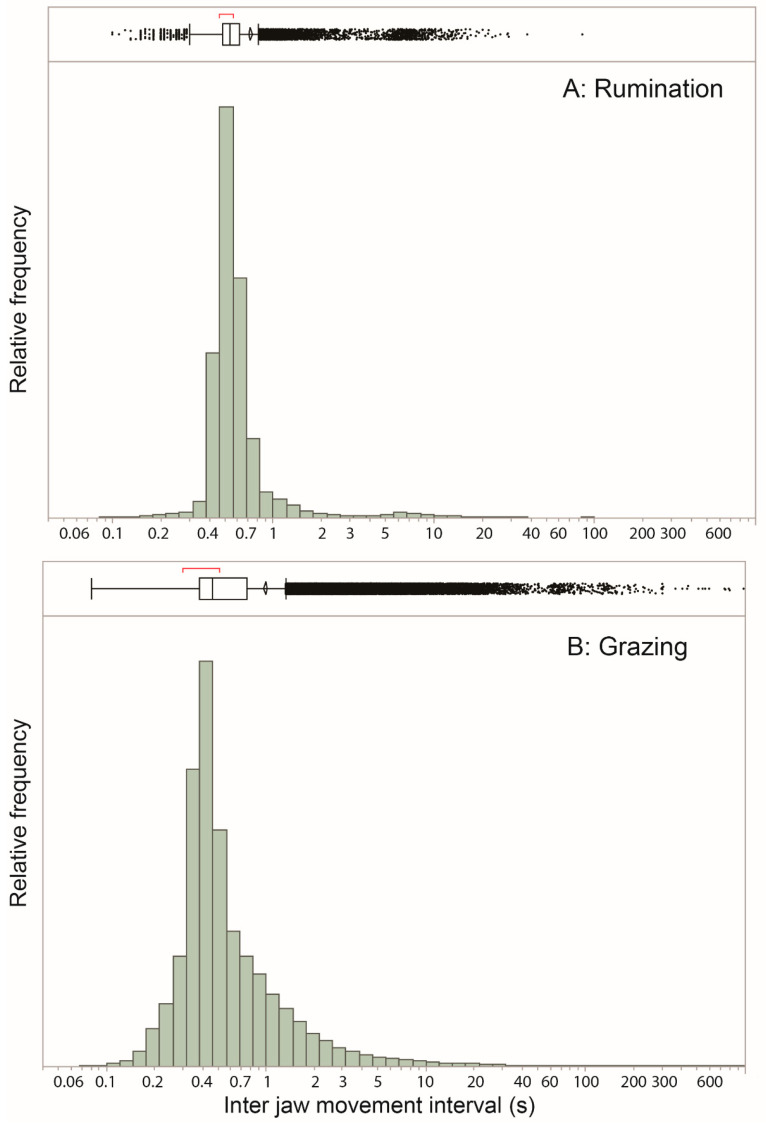
Frequency distribution of the inter-jaw-movement interval (s) for (**A**) rumination and (**B**) grazing, with logarithmic scaling. The extreme low end of the distribution may be due in part to false positive identifications of jaw movements. In the outlier box plots, the box center line is the median; box ends are 1st and 3rd quartiles; whiskers extend from box ends to the outermost value within the 1st/3rd quartile –/+ 1.5 × interquartile range; the center line of the confidence diamond is the mean and the lower and upper 95% of the mean (the left and right points of the diamond); and the bracket above box is the shortest half—the densest 50% of observations.

The frequency distribution of the inter-jaw-movement interval values designated as rumination is best viewed on a logarithmic scale, as shown in Figure 4A. Not unexpectedly, the vast majority of values are concentrated near an interval of ≈0.5 s, which corresponds to a normative within-bolus RJM of 120 min^−1^. But there is an important, albeit much smaller, secondary concentration of values at ≈7 s, evident also in the outlier box plot. This represents the inter-bolus interval, an essential feature in the identification of rumination bouts, but numerically scarce for obvious reasons. There was a total of 100 rumination bouts (i.e., continuous runs of rumination designation, bounded by non-rumination), the longest of which contained 3892 jaw-movement events (chews), and the shortest, 69 events. The number of jaw movements performed per bolus was approximately 50, although we did not rigorously annotate the bolus level. The median number of events per rumination bout was 410, or ≈8 boluses. Rumination accounted for <10% of total jaw movement events detected during the limited hours of the foraging routes (Table 1).

### 3.5. Grazing Jaw Activity

The non-rumination sections of the event-based plots, which are referred to collectively as “grazing” (with no definition for resting or travelling), did not reveal any rhythms or patterns of jaw activity that stood out the way rumination did. Here too, the frequency distribution of interval values is best viewed on a logarithmic scale (Figure 4B). This shows a concentration of values in the range 0.3–1 s, with a high proportion of values of ≈0.4 s, a small proportion of values exceeding 10 s, and a global maximum less than 1000 s. The most prominent feature in most of the 500-event plots was the preponderance of values of ≈0.4–0.5 s, especially because they often occurred in short bursts, rather than randomly. However, the entirety of interval values formed a fairly diffuse cloud of points without visually apparent repeating patterns or periodicity. There were, however, segments of sustained jaw activity with few deviations from the “baseline” interval of ≈0.5 s, which could be characterized as “intense” grazing in the behavioral sense (not vis-à-vis the vegetation).

**Figure 5:**
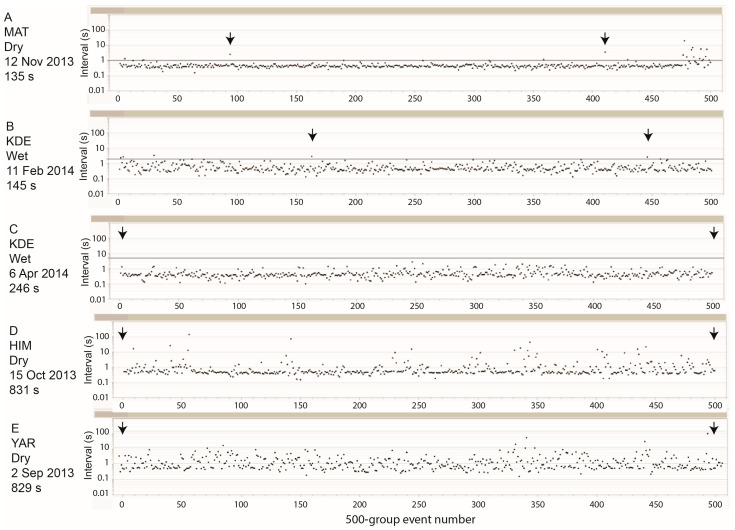
Event-based plots of grazing (non-ruminatory) jaw activity showing different patterns and degrees of intensity. The herd ID, season, date, and time interval between the two arrows are indicated. Panel (**A**) shows a run of at least 100 consecutive jaw movements with an inter-jaw-movement interval <1 s (horizontal line); in panels (**B**,**C**), the threshold was increased to 2 s and 5 s, respectively; panel (**D**) shows short segments bounded by intervals >5 s; panel (**E**) shows a highly erratic pattern of intervals.

**Figure 6:**
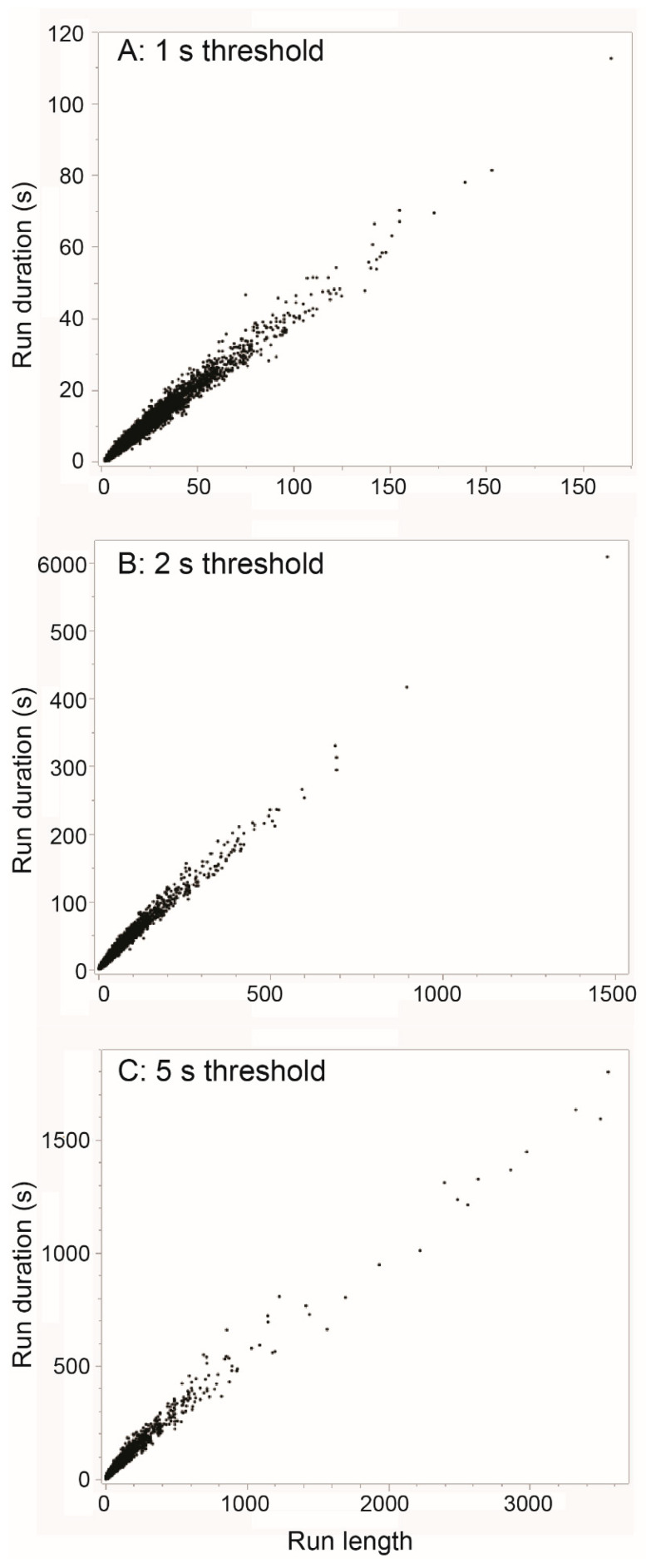
The relationship between run duration (i.e., sum of intervals) (s) and run length (i.e., number of intervals) (-) for three thresholds of the inter-jaw-movement interval: (**A**) 1 s; (**B**) 2 s; (**C**) 5 s. Note that the panels are independently scaled.

We used the length of sustained runs of sequential interval values that are below a defined threshold as an indicator of intensity. The minimum number of intervals required for counting as a sustained run was set, somewhat arbitrarily, to 100. When the threshold was 1 s, which is not so stringent given that it is approximately double the estimated baseline value, there were just 46 runs of at least 100 interval values < 1 s, over the 12 monitored routes (<4 runs per route, on average), and they accounted for ≈2% of all grazing-designated intervals. An example of such a run is shown in Figure 5A, which illustrates that even this most intense (and uncommon) level of jaw activity does not reach the rhythmicity and regularity displayed by the jaw activity of within-bolus, ruminatory chewing (Figure 3E). If we consider the total population of runs containing intervals < 1 s, the “baseline” interval value can be estimated from the relationship between the run duration (sum of intervals) and run length (number of intervals; Figure 6A). The slope of the almost-linear relationship is 0.43 s (≈140 JMs min^−1^).

When the tolerance threshold was increased to 2 s, the number of runs of at least 100 consecutive jaw movements increased to 499 (42 runs per route, on average), and 30% of all grazing-designated jaw movements were members of such runs. The interjection of intervals that are double or triple the characteristic interval of the baseline rhythm (≈0.5 s) results in a highly irregular rhythm (Figure 5B). We attribute the lack of rumination-like rhythmicity, even over short runs, to different time requirements of a jaw movement associated with the removal of a bite versus mastication of material in the mouth. This is expected to be more pronounced when the animal browses woody vegetation, which may impair the ease of prehension, and/or entail use of the split upper lip to perform rapid manipulative movements, which incur a time cost. When we considered the total population of runs containing intervals <2 s, the slope of the relationship between run duration and run length increased to 0.48 s (Figure 6B). Due to slight concavity of shape, the initial rising section of the relationship (sequence length < 200) had a higher slope (0.53 s).

When the threshold was increased to 5 s, the length of runs could reach many hundreds of interval values and even thousands (Figure 5C). Runs of at least 100 consecutive jaw movements <5 s contained 68% of all jaw movements. The relationship between the run duration and run length was moderately concave (Figure 6C); a linear slope approximation encompassing all sequence lengths yielded a value of 0.53 s, whereas the initial slope at a low sequence length (sequence length < 300) was 0.67 s. After excluding run sequences longer than 100, the remaining segments, which are bounded by an interval value > 5 s at each end, were usually dominated visually by a stream of values of ≈0.5 s that is interrupted by irregularly spaced eruptions of relatively long intervals (Figure 5D). But there were also segments of highly erratic intervals in which a dominance at ≈0.5 s was barely apparent (Figure 5E).

### 3.6. The Rate-Based Perspective

**Figure 7:**
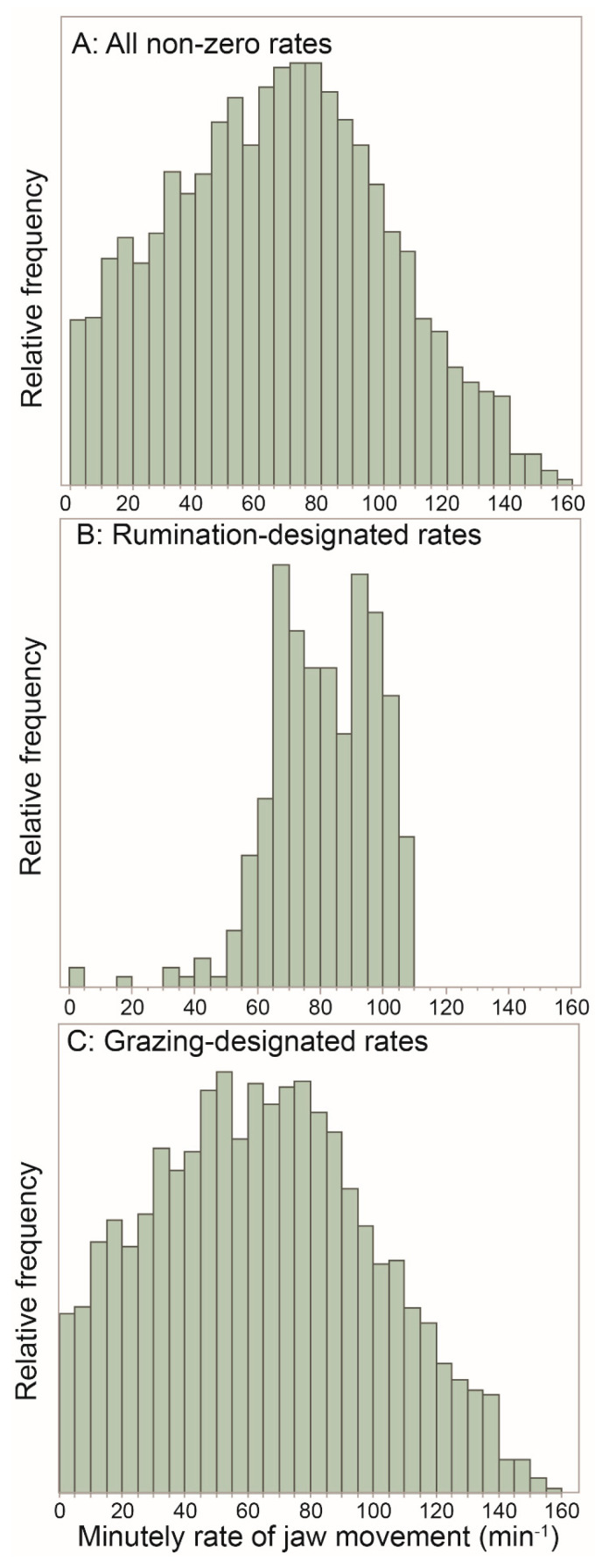
The frequency distribution of non-zero, minute RJM for the following: (**A**) the pooled dataset containing both rumination (including uncertain rumination) and grazing (*n* = 5096, median = 66 JM min^−1^); (**B**) the dataset containing rumination (including uncertain rumination) only (*n* = 359, median = 81 JM min^−1^); (**C**) the dataset containing grazing only (*n* = 4737, median = 64 JM min^−1^).

The diversity of patterns in jaw movement activity apparent in the event-based plots would be expected to give rise to a broadly shaped frequency distribution of the minutely rate of jaw movement. There was no consistent pattern in RJM with time into a foraging route. When all RJM values are viewed together, the frequency distribution is dominated by zero-RJM values, which alone constitute 6% of the dataset, and will be considered below (Section 3.7). For all *non-zero* RJM values, be they designated rumination *or* grazing, the frequency distribution is indeed broadly shaped, although not symmetrically so, and has a strong central tendency in the region of the median (66 JM min^−1^; Figure 7A). Below the median, there is a sizeable representation over the entire range down to almost zero, whereas above the median, the distribution tapers off completely at a maximum of 159 JM min^−1^. The frequency distribution of RJM values for minutes designated as rumination only (there were no 1 min-level designations of possible rumination) showed a more narrow-ranged and steeply-sided shape (median = 81 JM min^−1^; Figure 7B). This matches the almost mechanical jaw-movement rhythm associated with rumination. Some low RJM values are present and may seem unexpected. But rumination was assigned to JM events at the bout level, and bouts would usually include short pauses and interruptions in the jaw movement stream, which would depress RJM for the minute-slices containing them. Likewise, RJM values at the start and finish of a rumination bout are liable to be depressed when not aligned with the 1 min slices.

The frequency distribution of non-zero RJM values with the alternative designation of grazing (Figure 7C) was very similar to that of the pooled data and had a similar, but slightly lower, median of 64 JM min^−1^, considerably lower than the value of 81 JM min^−1^ for rumination. Given that one-third of RJM values shown exceeded 81 JM min^−1^, it is clear that the animals were able to sustain episodes of intense grazing that operate at a near-potential jaw movement rate, despite the ostensibly less-than-ideal foraging conditions of the Mediterranean woodland. But another third of RJM values were less than 48 JM min^−1^, lending credence to the notion of behavioral grazing intensity.

### 3.7. The Interval-Based Perspective

**Figure 8:**
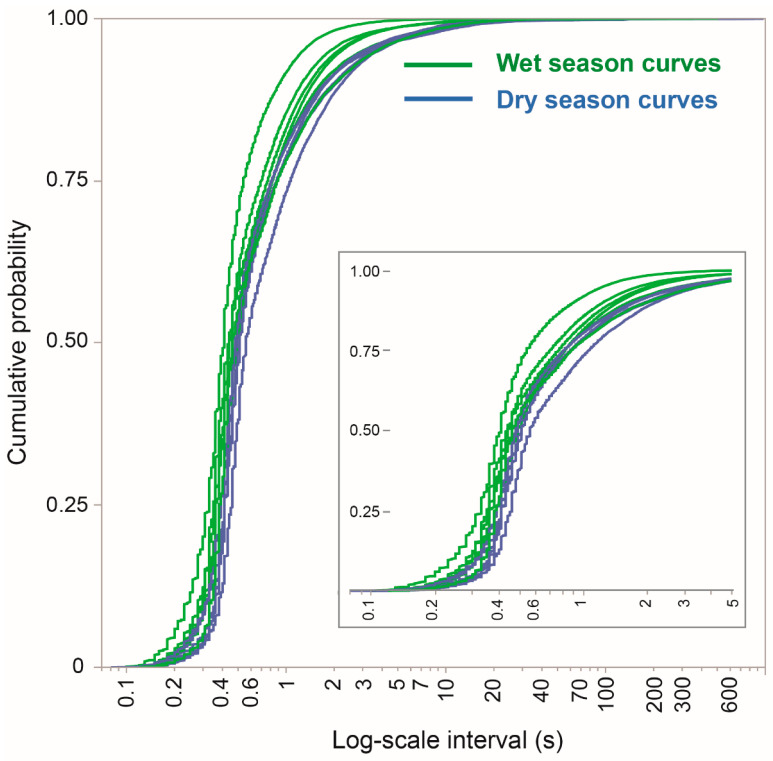
The CDF of inter-jaw-movement intervals designated as grazing (i.e., not rumination), for individual foraging routes. The interval is on the logarithmic scale. The insert shows an enlargement containing the interval range of 0.1–5 s.

In terms of the mechanistic resolution at which we are able to examine the grazing process, minutely rates hide a finer internal structure and also create a misfitting sub-population of zero values encountered above. Revisiting the interval values shown in Figure 4B, but presenting them as CDFs, addresses both of these issues and enables variability among seasons and foraging routes to be visualized (Figure 8). In terms of their broad features, the interval CDFs were similar across all foraging routes. Nevertheless, there was some degree of separation between the seasons, with the wet-season curves rising more sharply than the dry-season curves, meaning a lower proportion of the longer intervals. A detailed view of the region near an interval of 0.4 s (Figure 8 insert) suggests that the seasonal effect starts with the timing of events at this fine time scale.

**Figure 9:**
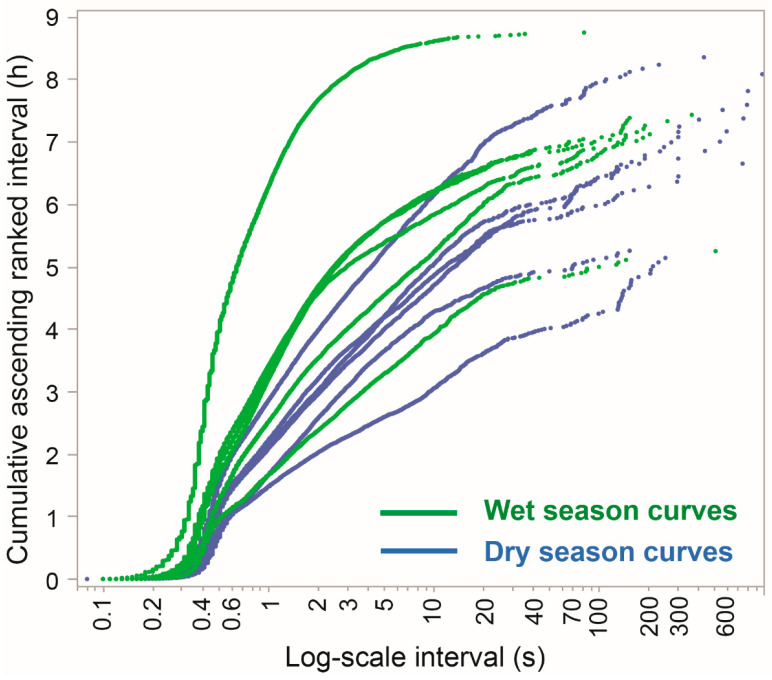
Empirical time accumulation curves showing the cumulative ascending ranked interval of jaw activity designated as grazing (i.e., non-rumination), for the 12 foraging routes. The interval is on the logarithmic scale.

A companion way of visualizing the distribution of inter-JM intervals is to show the contribution of the inter-JM interval to the foraging time, considering both its magnitude and its frequency in the data. To achieve this, the cumulative probability is converted into absolute time units by using the empirical time accumulation curve, equivalent to the cumulative ascending ranked interval (Figure 9). This essentially weights each interval by its own size, such that 100 1 s intervals give rise to the same vertical displacement on the time accumulation curve as a single interval of 100 s. The highest point on the curve shows the total grazing time over an entire foraging route, and the steepest sections of the curve correspond to intervals that contributed the most to the grazing time. Note how the scarcer upper end of the interval distribution of Figure 8 (>10 s, approximately) accounts for a substantial proportion of the time allocation in Figure 9. This perspective also shows a much stronger separation of foraging routes and a weaker separation of curves by season.

A further noteworthy feature of the time accumulation curves of Figure 9 is the two inflection points in the low-interval range of <1s. The first, at ≈0.4 s, corresponds to the bulk of intervals that are in the region of the baseline value, corresponding to the unconstrained, potential rate of jaw movement. A second inflection point occurs at ≈0.6 s, more strongly in some curves than in others, and connects to a less steep and relatively linear section of the curve, representing active grazing studded with logarithmically scaled pauses, interruptions, and breaks. These, we surmise, are due to constraints imposed by the vegetation via the search component of grazing or caused by the animal’s elastic entrainment to movement at the herd/shepherd level. As an indication of how limiting the foraging environment is, the sum total of time along a foraging route spent at a near-potential jaw movement rate is only ≈1 h, whereas sub-potential rates containing intervals as large as ≈30 s account for the bulk of a foraging route. There is some indication of a third region of inflection at ≈30 s, above which the time accumulation curves rise less steeply.

## 4. Discussion

### 4.1. Grazing Time

It is an open question as to whether there is a meaningful broken-stick structure to the time accumulation curves of Figure 9 in the region of 30 s or possibly 100 s. Such an inflection might imply a behavioral transition from ingestion-oriented behavior, with its attendant interruptions for search and locomotion, to non-grazing, which could be related to travel-oriented behavior. But we advise that caution be exercised to avoid over-interpretation of the curves, not least as this pattern was not evident in all of them. It may be preferable to view the time accumulation curves in their entirety, as a single continuum of intervals that scale logarithmically, but with an internal structure, rather than set what is ultimately an arbitrary threshold by which to define grazing time.

This approach views the animals as having engaged in grazing for the entire duration of the foraging routes, but in a way that incorporated logarithmically scaled breaks and interruptions in the baseline interval. That baseline interval generates the intrinsic unconstrained rhythm of jaw movement activity typical of grazing in a food-saturated environment. Note that at a finer time resolution, it is clear from the event-based plots that the baseline interval (i.e., unitary cycle time) is shorter for ingestive chews than for ruminatory chews. Similarly, it is clear from listening to the recordings that the unitary cycle time of a bite action is longer than that of either type of chew. It is further plausible that the time required for bite formation might be slightly longer when browsing woody vegetation than when grazing herbaceous vegetation, but such a determination is beyond our current scope. The extreme contrast between the wet and dry seasons in terms of vegetation conditions, and most notably the lower abundance and quality of herbaceous vegetation in the dry season, shifts the balance between herbaceous and woody vegetation in the diet [16] and would contribute to any seasonal deflection of the curves shown in Figure 8 and Figure 9.

Are the rates of jaw movement obtained in the present study consistent with published values? There is a paucity of comparable data. The most comprehensive quantitative review of ingestive behavior of grazing ruminants is provided by Boval et al. [20]. Based on the subset of studies reviewed that provided data on the jaw movement frequency of small ruminants, the average rate was 131 min^−1^ (range 92–171 min^−1^). These studies were in fact on sheep, and the vegetation was abundant grass or legume pasture (or microsward) [21,22,23]. Nevertheless, these results are at least consistent with the two estimates of the potential (unconstrained) jaw movement rates of 120 and 140 JMs min^−1^ derived earlier (Section 3.4 and Section 3.5) from the interval data. But note that the overall average RJM in the present study was approximately half this rate (Section 3.3), which we ascribe to search–locomotion–travel.

### 4.2. Grazing Intensity

Although the animal is considered to be grazing “all the time”, it is possible to give a quantitative expression to the *intensity* with which grazing activity is sustained or, conversely, the degree to which a particular foraging environment constrains intake. As seen in Figure 5A, animals *were* capable of sustaining jaw activity at the baseline interval, albeit for limited periods of time. The product of the total number of (non-rumination) JMs performed (NJ) and the “baseline” inter-JM time interval of unconstrained (ingestive) jaw activity (BT) gives the theoretical minimum grazing time, clean of any breaks and interruptions. That can be expressed relative to the potential grazing time (PT), i.e., the total duration of the foraging route minus rumination (and uncertain rumination) time or, equivalently, the sum of the grazing-designated intervals. A quantitative expression of grazing intensity is then GI = (NJ × BT)/PT. Equivalently, this can be defined as the ratio of actual to potential jaw movement rates, where the potential rate is the reciprocal of BT. The baseline interval BT was estimated for each foraging route as the peak of the frequency distribution of interval values. This was 0.4 s for nine of the routes and 0.6 s for the remaining three. An average value of 0.45 s was adopted. All but one of the GI values obtained were in the range of 0.31–0.55 (Table 1). There was one route with an exceptionally high GI of 0.84, showing that animals can in fact attain values approaching unity. The result was not artefactual; the soundtrack, the waveform, and the interval-based plots all supported a baseline interval in the region of 0.4 s, as for all other foraging routes. Had there been erroneous double-counting by the algorithm, a different or dual baseline would have appeared.

Various factors would be expected to introduce variation into GI, including the type of herd (dairy versus meat), the season (wet versus dry), the type of vegetation typically encountered by each herd, the specific vegetation encountered along the routes followed, an animal subject effect, including the physiological status, and between-animal variation in the baseline interval itself. The amount of data gathered would need to increase by an order of magnitude to separate them out; however, this is not currently feasible. Our impression based on direct observation of all the monitored herds over entire foraging routes is that the most important factor that determines the ability of the animal to sustain jaw activity is the availability of acceptable vegetation along the animal’s foraging route. Secondarily is the ease of prehension of the selected vegetation in the course of bite formation. This may differ between different woody species and between woody and herbaceous vegetation as a whole [24].

### 4.3. Implications for Mapping Grazing Impacts

From the point of view of the vegetation, if the ultimate goal is to conduct a balance between accretion and depletion processes at the landscape scale, a unit of animal presence needs to be converted to biomass offtake. All other things equal, *in particular* bite weight and chewing requirements, we would expect biomass offtake to scale monotonically with the grazing intensity. If the primary factors determining grazing intensity can be identified and mapped, it can be used as a weighting factor to improve the estimation of offtake.

As a first approximation at converting JMs to intake, we use the equation derived in the meta-analysis of Boval and Sauvant [25], which predicts that ≈46 JMs are expended per g intake, with modest downward adjustment with an increasing bite weight. Dividing total non-rumination JMs by this coefficient yields an average offtake per foraging route of ≈600 g. This appears to be a low value, although not implausible. In a somewhat comparable foraging environment, estimates of the daily intake of small ruminants foraging mixed herbaceous/woody vegetation [26] were in the range of 1410–2370 g DM, assuming an animal body weight of 50 kg. But note that the bite frequencies observed in that study were centered in the range of 50–60 *bites* min^−1^. Contrast that to the present study, in which *RJM* associated with grazing was 60 min^−1^, including both bites *and* chews. Boval and Sauvant [25] estimate the chew-to-bite ratio in small ruminants to be ≈1.8, which implies a bite frequency of ≈21 min^−1^ or one-third of that observed in [26]. That is in agreement with the bite rates observed in goats foraging Mediterranean forest rangeland [27], being 17.7, 21.9, and 22.1 min^−1^ in the spring, summer, and autumn, respectively. In that study, goats were determined to be in a large energy deficit in the summer and autumn seasons. These findings are consistent with our estimate of intake being so low.

One upshot of lower grazing intensities is that more hours of foraging are required to accumulate the same “net” hours of grazing (as a surrogate for intake). This could be taken into consideration in decisions related to grazing management and herd supplementation. Some of the meat-breed herds in this study practiced extremely long foraging routes, returning after dark even in the summer months [5]. This seemed unnecessarily long to harvest daily requirements but is rational if the vegetation being exploited supports/induces low GI values, which was mostly the case.

## 5. Conclusions

Acoustic monitoring is a promising methodology for studying herbivore grazing at a mechanistic level. At minimum, it can generate the timeline of unclassified sound bursts generated by jaw movements that perform bite or chew actions. Compared to classifications into bites, chews, and chew-bites, this timeline is easier to generate automatically and can achieve greater accuracy. The algorithm used to detect and timestamp all sound bursts that contained chew or bite sounds, *without* classifying them, performed well in terms of the accurate identification of true jaw movements while ignoring the sound bursts generated by extraneous noise, as could be identified aurally.

In terms of characterizing how the animal is interacting with the foraging environment, operating at the jaw-movement level enables grazing to be profiled in a highly sensitive way, as seen in the empirical time accumulation curve. This portrays the fine structure of grazing behavior in terms of the size of the interval between consecutive jaw-movement events, with no definition of grazing time in the conventional sense. As a quantitative measure of the animal–vegetation interaction, we defined grazing intensity as the theoretical minimum time required to perform the number of jaw movements counted along a foraging route, relative to the route’s duration (ignoring rumination). This could be used to refine how grazing impacts are computed and for gauging how well animal requirements are being met.

After the exclusion of rumination bouts, which have a highly distinctive signature pattern of jaw movements, jaw activity associated with eating populated the entire foraging route, but in a highly uneven way. The timeline of jaw movements could be described as comprising a baseline rhythm of unconstrained jaw activity (typical of a food-saturated foraging environment), which is shattered with interruptions for which the duration scales logarithmically. We presume the primary driver of those interruptions to be related to the search phase of the intake process, as dictated by the abundance, structure/architecture, and nutritional quality of the vegetation. The JM timeline afforded by acoustic monitoring contains a wealth of information not conveyed by a simple binary distinction between “grazing” and “not-grazing” states and possibly even by bite rate.

## Figures and Tables

**Table 1:** Summary features of the 12 foraging routes monitored acoustically. The column “All JM Events” includes rumination and uncertain rumination (Rumin?) jaw-movement events. Grazing intensity (dimensionless) is derived in Section 4.2.

Season	Date	Herd Name and Code	Start Time (hh:mm)	End Time (hh:mm)	Duration (hh:mm)	Duration (s)	All JM Events	Median Interval (s)	Maximum Interval (s)	Grazing JM Events	Rumination JM Events	Rumin? JM Events	Grazing Intensity
Dry	2 September 2013	Ya’aran (YAR)	14:25	19:45	05:20	19,200	15,425	0.56	154	15,425	0	0	0.37
	15 October 2013	Himelfarb (HIM)	06:48	12:45	05:57	21,420	13,366	0.49	253	13,366	0	0	0.32
	23 October 2013	Refaim (REF)	10:27	17:41	07:14	26,040	22,591	0.53	740	20,462	1856	273	0.38
	29 October 2013	Kedoshim East (KDE)	08:05	16:31	08:26	30,360	23,657	0.53	560	19,256	3375	1026	0.32
	31 October 2013	Kedoshim West (KDW)	07:15	16:25	09:10	33,000	24,742	0.54	975	20,369	4373	0	0.31
	12 November 2013	Mata (MAT)	07:00	15:30	08:30	30,600	27,206	0.49	432	26,706	500	0	0.40
Wet	25 December 2013	Ya’aran (YAR)	11:40	17:00	05:20	19,200	16,284	0.46	508	16,018	0	266	0.38
	14 January 2014	Mata (MAT)	07:50	15:40	07:50	28,200	25,054	0.51	365	21,882	2856	316	0.37
	30 January 2014	Kedoshim West (KDW)	07:55	16:20	08:25	30,300	38,118	0.47	203	31,365	6753	0	0.55
	11 February 2014	Kedoshim East (KDE)	09:40	17:10	07:30	27,000	32,099	0.46	117	29,651	1370	1078	0.53
	20 February 2014	Refaim (REF)	09:05	17:00	07:55	28,500	34,304	0.46	153	31,487	2817	0	0.53
	6 April 2014	Kedoshim East (KDE)	09:50	19:10	09:20	33,600	61,736	0.41	81	58,707	2368	661	0.84
		All merged			90:57	327,420	334,582	0.48	975	304,694	26,268	3620	0.45

## Data Availability

Data will be made available on request.

## Abbreviations

BT = baseline inter-jaw-movement time interval; CDF = cumulative distribution function; GI = grazing intensity; GIS = geographic information system; GPS = global positioning system; JM = jaw movement; NJ = number of jaw movements performed; PT = potential grazing time; RJM = rate of jaw movement.
